# Case of Frontal Glioma With a Factitious Disorder of Self-Inflicting Dental Injuries Managed by Coronally Advanced Flap With Orthodontic Buttons

**DOI:** 10.7759/cureus.43602

**Published:** 2023-08-16

**Authors:** Ashis Pattnaik, Naina Pattnaik, Monalisa Das, Debasish Dash

**Affiliations:** 1 Neurosurgery, All India Institute of Medical Sciences, Bhubaneswar, IND; 2 Periodontology, Hi-Tech Dental College and Hospital, Bhubaneswar, IND; 3 Orthodontics and Dentofacial Orthopedics, Hi-Tech Dental College and Hospital, Bhubaneswar, IND; 4 Orthodontics and Dentofacial Orthopedics, Meghna Institute of Dental Sciences, Nizamabad, IND

**Keywords:** glioma, orthodontic buttons, coronally advanced flap, gingivitis artefacta, factitious disorder

## Abstract

Factitious disorders represent deliberately fabricated dissimulation of physical and psychological signs and symptoms seeking medical attention by the patient. Usually, they are ignorant of conventional treatment and consistently change their version of signs and symptoms. Due to various changes in the version, they do not respond to the treatment. They describe their signs and symptoms as dissimulated, imaginative, and exasperated, involving any part of the body. Gingivitis artefacta is an unusual and dramatic presentation with self-inflicted physical injury to the gingival tissues. We present an extremely rare case of frontal lobe glioma causing abnormal psychology of factitious disorder resulting in self-inflected injury to gingiva in an adult male. This case also highlights the management of the dental condition of multiple recessions with coronally advanced flaps with orthodontic buttons.

## Introduction

Factitious disorder patients present with unusual dramatic signs and symptoms inconsistent with any pathology or disease. They are highly anxious, seeking attention, and ready to undergo any type of investigation. This leads to suspicion in the clinician’s mind toward psychiatric involvement. In factitious disorder, the signs and symptoms of the patients are either fabricated or dissimulated aiming toward being ailing always and thus dragging attention [1]. They show dramatic or feigning physical or psychological symptoms. They confuse the clinician and restrain from any definitive treatment. They usually do not respond to usual medical or surgical treatment.

Clinically, it is very difficult to diagnose such a disease because of fabricated history or continuous changes in the version of the patient. After resolving one symptom, there may be an emergence of new symptoms [2]. Other names of this disease are factitious disorder, Munchausen’s syndrome, Hospital hopper syndrome, thick chart syndrome, and black hole patients [3]. According to medical expertise, it is one of the most challenging disorders to diagnose [1]. With due course of time and enhancement in our medical science, the incidences of factitious disease are also increased [3].

The various signs and symptoms of the factitious disorder such as neurological, gynecological, urological, dental, and others were well documented [4]. Oral manifestations of self-inflicting dental injuries include ulcerations, lacerations of the tongue, and buccal mucosa due to biting and scratching of the gingiva (gingivitis artefacta) resulting in gingival recession associated with the bone loss [5-7]. There are various traumatic gingival injuries such as thermal injuries, and chemical or physical injuries have been well documented [6,7]. Further, the physical injuries to the gingiva either due to accidental or iatrogenic causes depict an intentional self-abrasion of the gingival tissues caused by the patient’s own fingernail or use of any toothpick or pin. But dentist often faces challenges while diagnosing and managing such case because artefacta represent similar clinical presentation with other oral lesions.

Here, we present a case of self-inflected injury to the gingiva by an adult male with abnormal psychology caused by a structural lesion in the frontal lobe of the brain. This case also highlights the management of multiple recessions with coronally advanced flap with orthodontic buttons.

## Case presentation

Clinical presentation and imaging

**Figure 1:**
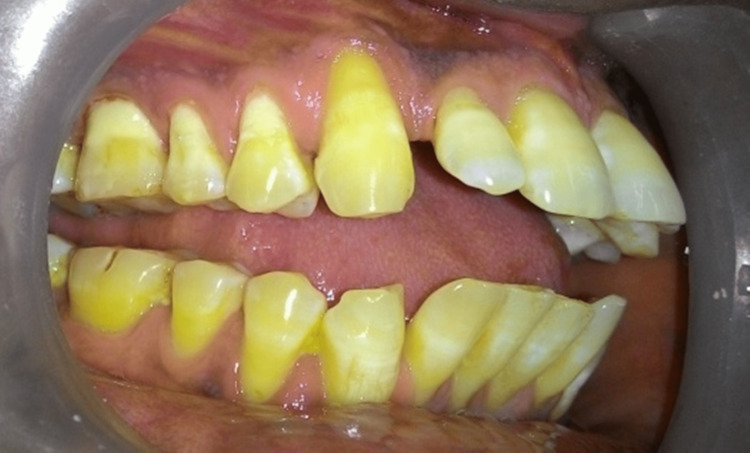
Millar class I gingival recession in relation to tooth no 13, 14, and 15 Millar class I gingival recession in relation to tooth no 13, 14, and 15.

**Figure 2:**
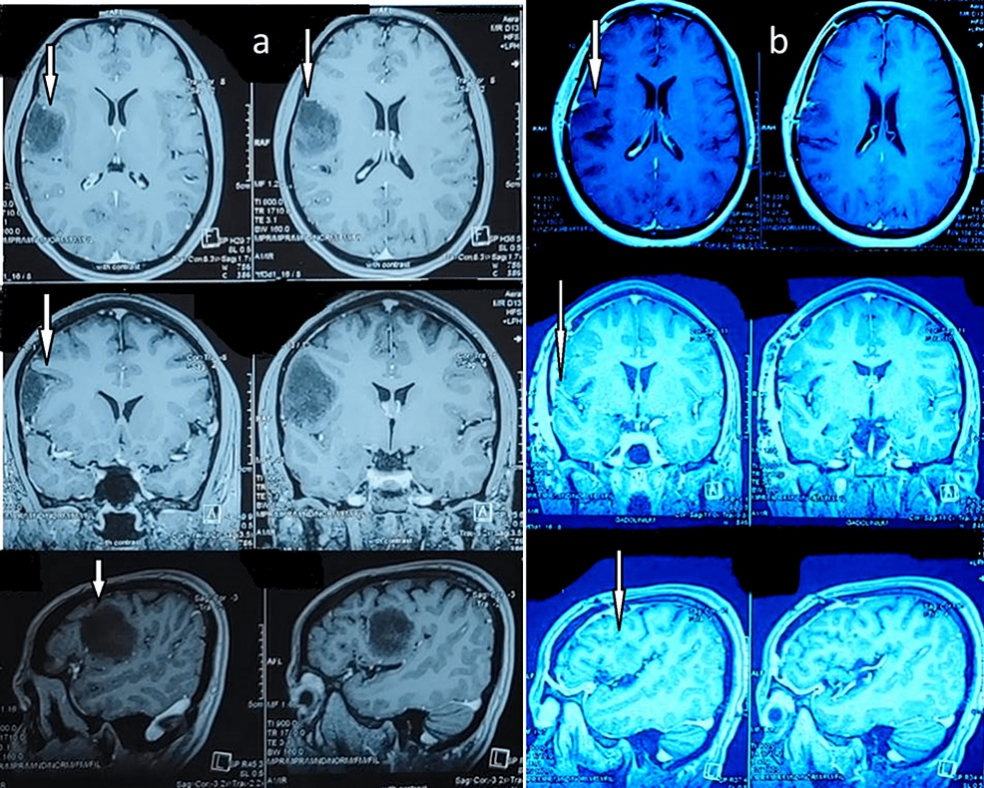
Brain MRI (a) Pre-operative brain MRI with contrast showing a non-enhancing space-occupying lesion in the right posterior frontal lobe. (b) Post-operative image showing complete excision of the lesion. The arrow marks show a brain MRI with contrast showing a space-occupying lesion in the right posterior frontal lobe. (b) Post-operative image shows complete excision of the lesion.

A 39-year-old male reported to us with chief complaints of bleeding from gums and sensitivity in maxillary teeth for the past four years. He had already visited many dental clinics and had undergone symptomatic treatment. He looked dull, scrawny, and thin. He complained of headaches frequently. There were multiple scar marks on his face, hand, and neck region. He was highly anxious and concerned about his dental problem. On clinical examination, there was Millar class I gingival recession in relation to tooth no # 13, 14, and 15 (“FDI notation” or “ISO-3950 notation”) (Figure 1). There was a loss of papilla height on the distal aspect of the right maxillary canine with moderate growth in gingival size in that particular region. There was also the presence of fluorosis stain with limited plaque deposits. On further questioning, he showed a very unusual behavior of his extra-cautious nature toward his dental condition. He also gave a history of scratching of gingiva with a pen and a small sharp stick in that particular region (papillary and buccal). He was so inclined for cleaning his teeth that he brushes five to six times per day vigorously until bleeding occurs. This dragged to the suspicion of a medically unexplainable presentation of psychological precipitant. Based on clinical presentation and frequent doctor shopping and abnormal way of presentation of consciousness toward his dental condition, a diagnosis of gingivitis artefacta major was established and a psychiatric consultation was done. The psychological disturbances developed in the last year, and his marital status was also in trouble. From a psychiatric point of view, cognitive restructuring was done with a non-confrontational, sympathetic type of approach. After psychiatrist consultation, dental treatment such as scaling and root planning along with oral hygiene measures was started due to the presence of plaque and Miller class I gingival recession which occurred due to gingivitis artefacta major. However, the patient complained of more frequent and intense headaches; thus, neurological consultation was done. Brain imaging (MRI brain with contrast) showed an intra-axial space occupying a lesion on the right side of the posterior frontal region (Figure 2a). Simultaneously, psychiatric therapy was continuing. After clearance from the psychiatrist, brain surgery was planned.

Surgical procedure

**Figure 3:**
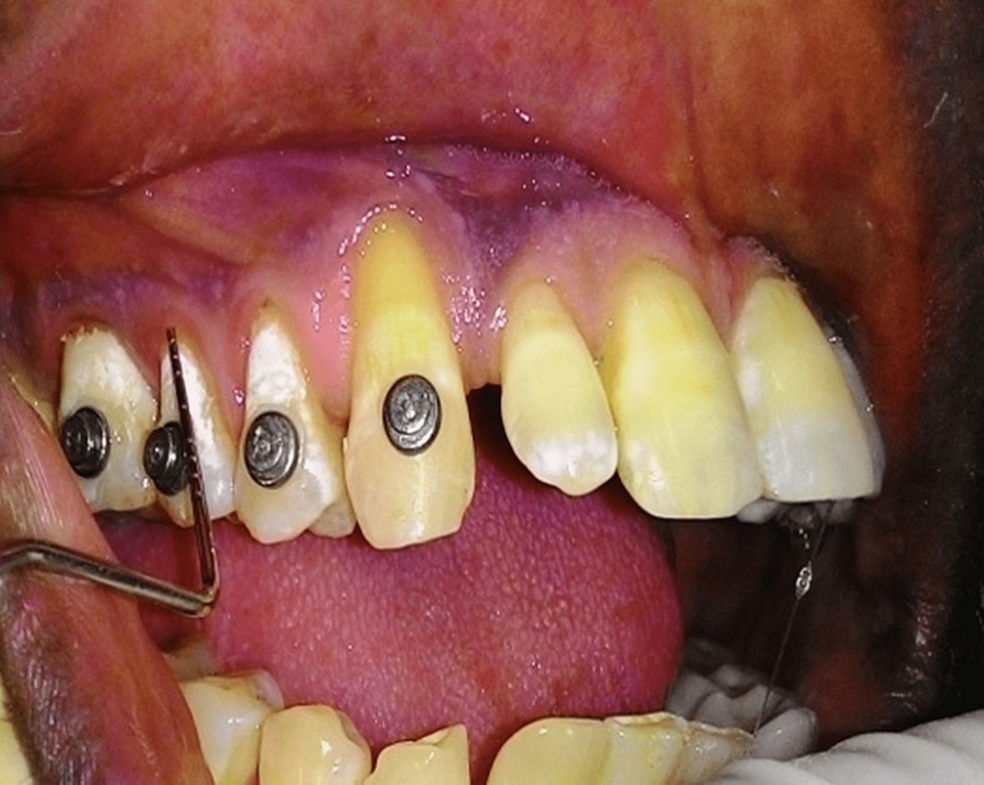
Orthodontic buttons on the facial surface of teeth

**Figure 4:**
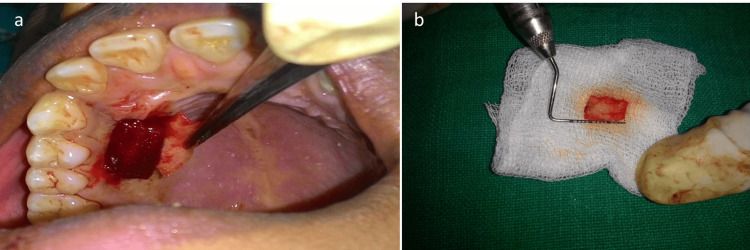
Trap door method and connective tissue graft harvested (a) Trap door method of collection of a connective tissue graft from the hard palate. (b)  Connective tissue graft harvested from the hard palate.

**Figure 5:**
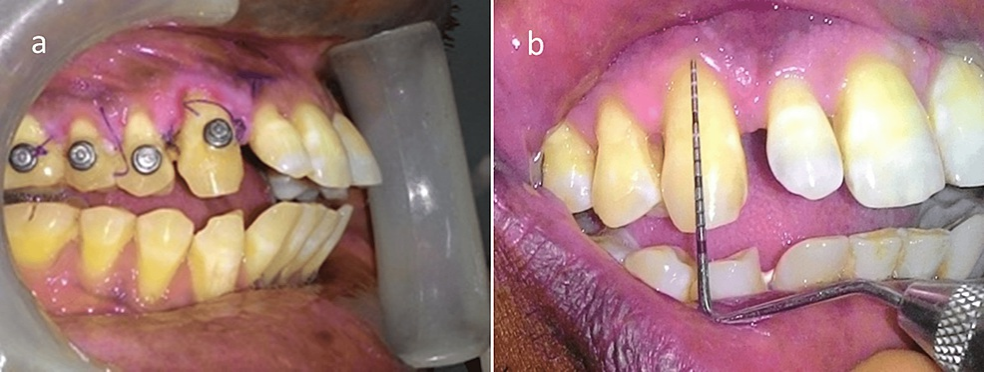
One week and six months post-operative (a) One week post-operative connective tissue graft take up. (b) Six months post-operative with increased attached gingiva.

**Figure 6:**
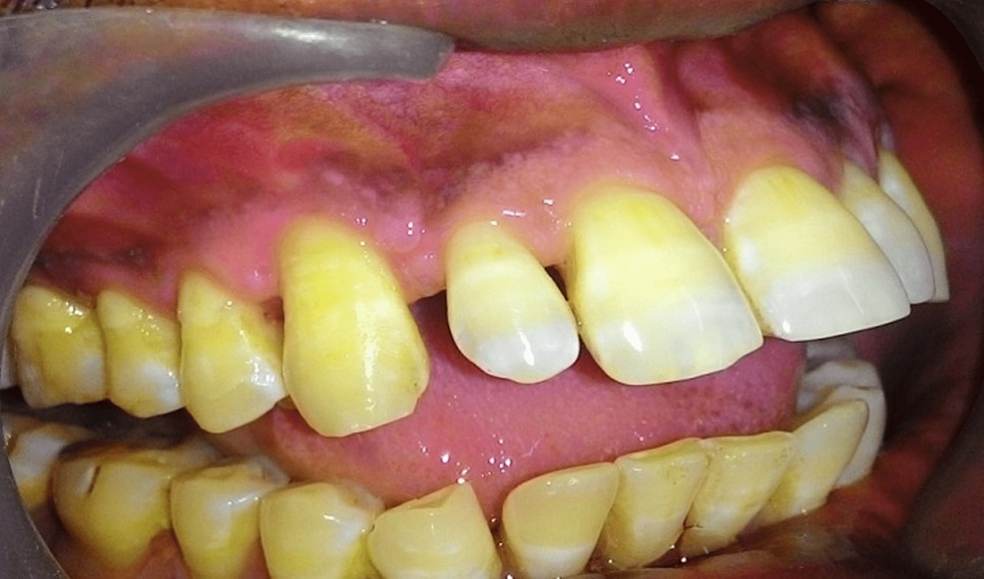
One year post-operative One year post-operative with an increase in clinical attachment loss (CAL) and keratinized width of attached gingiva.

A frontal craniotomy along with gross total excision of the lesion was done. Histopathology was suggestive of WHO grade 2 astrocytoma. After a follow-up of three months, the patient’s psychiatric symptoms completely subsided. The post-operative MRI brain showed complete removal of the tumor (Figure 2b). Following six months of brain surgery, further dental treatment including periodontal root coverage procedure was planned for multiple gingival recession. Orthodontic buttons were placed prior to surgery on the labial aspects of teeth (Figure 3). After local anesthesia administration, a horizontal incision was placed at the base of the papillae following de-epithelization. A full-thickness mucoperiosteal flap was raised to expose the root surface. For the donor site, an incision was given on the palate and a partial thickness flap was raised according to the size of the template. Following this, connective tissue was elevated (Figures 4a, 4b). The connective tissue was sutured to cover the exposed root surface. The full-thickness flap was coronally advanced and secured around the orthodontic buttons with suspended sling sutures (Figures 5a, 5b). All the chemical plaque control methods were activated for the patients. After one year, uneventful healing with complete root coverage was achieved with an increase in clinical attachment loss (CAL) and keratinized width of attached gingiva (Figure 6). There was no further injury to any part of the body according to the psychiatrist.

## Discussion

Deliberate fabrication and dissimulation of physical and psychological signs and symptoms seeking medical attention by a patient are clear indications of factitious disorder [5]. Usually, they are ignorant of conventional treatment and consistently change their version of signs and symptoms. Due to various changes in version, they do not respond to the treatment or new symptoms develop [5]. However, they are overwilling to be exposed to various investigations to gain medical attention. They describe their sign and symptoms to be dissimulated, imaginative, and exasperated involving any part of the body [6].

Gingivitis artefacta comes under the broad classification of gingival disease which is caused by self-inflicting injuries to gingival tissues. Damage to the gingiva can be done by impinging any foreign object or by scratching of gingiva with fingernails. This leads to ulceration, bleeding, baring of soft tissue, and exposure of underlying bone [7-8].

It is further subdivided into minor and major by Stewart (1976). Gingivitis artefacta minor is more commonly seen in the intraoral site and is the less severe form [9]. The etiologic factors are rubbing or pricking the gingiva using fingernails or abrasive food such as crisps [10]. The major form is more severe having a more bizarre configuration, affecting periodontal tissue, and may expose the underlying bone [10-11]. In the present case described, the lesions were consciously self-inflicted by the patients with the help of matchsticks. Gingivitis artefacta major is the physical manifestation of an underlying emotional disorder [7]. It is very difficult to diagnose and can be easily missed during routine examinations. There are always chances of recurrence of lesions and failure of periodontal treatment as they are associated with underlying abnormal psychology. Until the psychological treatment is corrected, the recurrence of the lesion or failure of treatment will occur. Differential diagnosis of gingivitis artefacta may include physical or chemical injury of the gingiva, aphthous ulcer, and gingival recession [7]. Pattnaik et al. described a case series of gingivitis artefacta major managed successfully with an interdisciplinary (periodontal and behavioral) approach [7].

In the present case, a brain tumor led to psychological and cognitive disturbances in the patient. This further leads to the development of consciously self-inflicted gingival injuries by the patient. Here, the gingival injuries were created with the help of pins and matchsticks and thus leading to severe gingival recession and bone loss.

Frequent visits to various dentists in the face of self-inflicted injury indicate the intention to gain medical attention. This is a clear example of a factitious disorder. A proper management is required keeping in mind factious disorder. Although the previous history of this case with collateral history from family and friends was acquired, it may not be corroborative. The primary aim of the management should be minimizing harm by curtailing unnecessary investigations and interventions. A cognitive restructuring along with addressing coping skills and emotional conflict therapy was implied upon the patient [10-11].

Brain tumors particularly involving frontal and temporal lobes are known to cause psychological and cognitive disturbances. Carmona-Bayonas et al. reported a case of hyperreligiosity in a case of frontal lobe tumor [12]. However, such typical presentation of brain tumor in the form of factitious disorder has not been reported in the literature. The association of progressively increasing headaches helped in the diagnosis of the causative lesion in the brain. Once the glioma involving the brain was removed, patients’ psychological disturbances completely improved with no further episodes of self-injury.

There are various different modalities for recession coverage such as free gingival autograft, lateral sliding flap, coronally advanced flap, and subepithelial connective tissue graft [13]. For multiple recessions, the coronally advanced flap is the most commonly used technique. The most challenging in this technique is the prevention of apical migration and securing the flap in position coronally to cemento-enamel junction (CEJ). The orthodontic buttons can be used in securing the flap in its accurate position with the help of sling sutures. Ozcelik et al. and Aroca et al. were the first ones to report this technique [14-15]. A complete root coverage was achieved by using this technique. The results were stable for three months, six months, and one year. The orthodontic button provides anchorage to the coronally advanced flap. The suspended sutures placed over the buttons gained maximum coronal advancement and prevented apical movement. The post-surgical follow-up of this case showed very stable and excellent results. There was complete root coverage with adequate keratinized width of the attached gingiva.

## Conclusions

This case highlights the extremely rare presentation of a frontal brain tumor (glioma) with a factitious disorder of dental gingival injuries caused by abnormal psychological disturbances resulting in self-inflicting injury. Organic structural lesions of the brain should always be ruled out before overburdening with psychological interventions. Psychological interventions should be primarily symptomatic before the primary cause of such disorders is found. Non-confrontational, sympathetic approaches, behavior modification, and positive reinforcement become boon for such cases. The present case also illustrates the value of dental surgical procedures like orthodontic buttons in multiple gingival recessions.
